# Anterior Spinal Artery Aneurysms Associated with Coarctation of the Aorta: A Focused Narrative Review with an Illustrative Case

**DOI:** 10.3390/jcm15197364

**Published:** 2026-09-22

**Authors:** Dong Sun Park, Young Il Chun, Hong Gee Roh, Jeong Jin Park, Yoo Sung Jeon

**Affiliations:** 1 Department of Neurosurgery, Changwon Hanmaeum Hospital, Hanyang University, Changwon 51139, Republic of Korea; hands17@hanmail.net; 2 Department of Neurosurgery, Konkuk University Medical Center, Seoul 05030, Republic of Korea; 3 Department of Radiology, Konkuk University Medical Center, Seoul 05030, Republic of Korea; 4 Department of Neurology, Konkuk University Medical Center, Seoul 05030, Republic of Korea

**Keywords:** anterior spinal artery, aneurysm, coarctation of the aorta, spinal subarachnoid hemorrhage, collateral circulation, endovascular treatment

## Abstract

Anterior spinal artery (ASA) aneurysms are rare causes of subarachnoid hemorrhage (SAH), and coarctation of the aorta (CoA) may recruit the spinal arterial circulation into a high-flow collateral network. We report the case of a 58-year-old woman who was resuscitated after pulseless electrical activity arrest. Noncontrast CT demonstrated SAH concentrated in the cervical spinal canal with limited intracranial extension. Cervically extended CT angiography identified a C5-6 ventral spinal aneurysm, severe thoracic CoA with extensive collateralization, and diffuse left subclavian artery hypoplasia. Digital subtraction angiography confirmed the presence of a multilobulated sidewall ASA aneurysm supplied by an artery of the cervical enlargement arising directly from the subclavian artery. Selective coil embolization achieved complete aneurysm occlusion with preservation of the parent artery; this was followed by C5-6 laminectomy and posterior arthrodesis the next day. At approximately 1 year, the patient had regained left-sided motor function, whereas severe right-sided weakness and wheelchair dependence persisted. A focused PubMed/MEDLINE review identified eight well-characterized previously published cases that included sufficient data for detailed tabulation of both a spinal arterial aneurysm and CoA. CoA-associated lesions clustered in the cervical and upper thoracic regions and were managed microsurgically, endovascularly, or conservatively, with no comparative evidence favoring one approach. Spinal-predominant SAH should raise suspicion for a spinal vascular source and prompt consideration of cervical vascular imaging. When enlarged spinal or systemic collateral vessels are identified, assessment of the thoracic aorta may also be considered.

## 1. Introduction

Aneurysms arising from spinal arterial circulation are rare and likely under-recognized. In a 2022 systematic review that identified 107 patients across 82 publications, the anterior spinal artery (ASA) was the most frequently involved arterial axis, and subarachnoid hemorrhage (SAH) was the most common presentation [1]. A subsequent two-center retrospective series and updated systematic review reported 208 isolated spinal aneurysms in 164 patients; 82% were fusiform, 90% presented with SAH, and spontaneous regression was documented in 52% of conservatively managed lesions [2]. Recent syntheses also show that microsurgery remains a major component of reported treatment, although standardized algorithms and comparative evidence are lacking [2,3,4,5].

These lesions are anatomically and mechanistically heterogeneous, ranging from saccular aneurysms to fusiform, dissecting, or pseudoaneurysmal dilatations, including lesions arising at the junction of a radiculomedullary artery with the longitudinal ASA. They may be isolated or associated with arteriovenous shunts, inflammatory or connective tissue disorders, pregnancy, mechanical or degenerative spinal pathology, or occlusive vascular disease that recruits the spinal arterial circulation as a collateral pathway [6,7,8,9].

Coarctation of the aorta (CoA) is a particularly relevant flow-related condition. The persistent pressure gradient across the obstruction promotes collateral recruitment through systemic and spinal arterial networks. When the ASA is incorporated into this collateral circuit, chronic flow augmentation and focal wall stress may contribute to aneurysm formation [10,11,12,13].

Here, we describe a ruptured cervical ASA aneurysm associated with previously unrecognized CoA in a 58-year-old woman who presented with cardiac arrest and spinal-predominant SAH. This case highlights early detection of the culprit lesion on the initial cervically extended CTA, angiographic characterization of bilateral collateral pathways, and staged coil embolization before cervical decompression. We also provide a focused narrative review structured by linked phenotype–mechanism and management frameworks, with a separate patient-level synthesis of CoA-associated cases.

## 2. Materials and Methods

### 2.1. Case Report and Review Design

The case report was prepared in accordance with the CARE guidelines [14]. The literature component was conducted as a focused narrative review using a structured PubMed search and a separate patient-level synthesis of CoA-associated cases.

### 2.2. Search Strategy

PubMed was searched from database inception through 31 July 2026. The general search used the following spinal arterial aneurysm block: (“spinal artery aneurysm” OR “spinal aneurysm” OR “anterior spinal artery aneurysm” OR “posterior spinal artery aneurysm” OR “lateral spinal artery aneurysm” OR “radiculomedullary artery aneurysm” OR “radicular artery aneurysm”). The CoA-focused search combined this block using AND with (“Aortic Coarctation” [MeSH] OR “coarctation of the aorta” OR “aortic coarctation” OR “aortic isthmus stenosis” OR “interrupted aortic arch”). To identify additional reports, reference lists and forward citations of eligible articles and key reviews, including the two principal quantitative syntheses [1,2], were screened.

### 2.3. Eligibility Criteria and Study Selection

Full-length primary clinical reports in any language were eligible if they described an aneurysm of the spinal arterial circulation and provided patient-level clinical, imaging, hemodynamic, or treatment data. Reviews were used only for contextual synthesis and identification of additional reports and were not included in the patient-level synthesis. Primary reports were excluded if they described aneurysms outside the spinal arterial circulation or non-aneurysmal spinal vascular lesions; conference abstracts or reports containing insufficient patient-level data were also omitted. The CoA-focused synthesis also required documentation of both a spinal artery aneurysm and CoA; cases associated with an arteriovenous shunt were excluded. For detailed tabulation, sufficient data were required to verify the aneurysm and CoA and to extract the lesion location, clinical presentation, and management. One reviewer screened the records; a second reviewer verified the extracted patient-level data. Discrepancies were resolved by discussion, and multiple reports describing the same patient were merged.

### 2.4. Data Extraction and Synthesis

Extracted variables included demographics, associated vascular conditions, aneurysm location and phenotype, presentation and rupture status, treatment, parent-artery preservation, surgical adjuncts, complications, and clinical or imaging follow-up. Data were verified against the original reports when available; information that could not be corroborated was recorded as not stated. The general evidence was organized using the author-defined phenotype and mechanism axes (Table 1a,b) and treatment framework (Table 2), whereas CoA-associated cases were synthesized separately at the patient level (Table 3). No de novo quantitative pooling was performed.

### 2.5. Case Data Sources

Clinical, imaging, and procedural information was obtained through a retrospective review of the patient’s medical records and imaging studies. Long-term neurological status was obtained from follow-up documentation.

## 3. Case Report

### 3.1. Presentation and Initial Imaging

A 58-year-old woman was found unresponsive at home and transported to the emergency department. Cardiopulmonary resuscitation was performed for pulseless electrical activity arrest, and return of spontaneous circulation (ROSC) was achieved prior to neurological evaluation. On initial examination after ROSC, the patient was drowsy and showed no voluntary movement in any extremity (Medical Research Council [MRC] grade 0/5 bilaterally).

Emergency noncontrast head CT demonstrated SAH predominantly involving the cervical spinal canal, with a relatively limited intracranial component. This spinal-predominant distribution raised suspicion of a spinal vascular source and prompted CTA of the head and neck, with inferior coverage extended to include the aortic arch.

CTA showed no culprit intracranial aneurysm. Instead, it demonstrated a focal aneurysmal lesion along the ventral spinal arterial axis at C5-6, which was spatially concordant with the predominant hemorrhage, as well as severe thoracic CoA and an extensive collateral network.

### 3.2. Magnetic Resonance Imaging

Cervical spine MRI obtained on the day of presentation demonstrated extensive spinal SAH extending through the cervical spinal canal to T4, with heterogeneous perimedullary signal and multilevel effacement of the perimedullary cerebrospinal fluid space. A focal ventral intradural signal void at C5-6 corresponded to the aneurysmal lesion identified on CTA. No definite intramedullary hemorrhage was identified. The initial CT, CTA, and MRI findings are shown in Figure 1.

### 3.3. Angiographic Findings and Hemodynamic Interpretation

Selective digital subtraction angiography (DSA) revealed a 9.8 × 6.9 mm multilobulated sidewall aneurysm of the ASA at C5-6, supplied by an artery of the cervical enlargement arising directly from the subclavian artery. No associated arteriovenous shunting was identified. Although no active contrast extravasation was observed during angiography, the aneurysm was considered the most likely source of bleeding because its cervical location was anatomically concordant with the spinal-predominant SAH and no culprit intracranial aneurysm had been identified on CTA. The attribution of rupture was therefore based on the combined clinical and imaging findings rather than direct visualization of active bleeding.

Left-sided cineangiography demonstrated sequential opacification of an enlarged cervical radiculomedullary vessel and a longitudinal spinal arterial channel, with caudal progression toward lower thoracic segmental outflow (Video S1). Right-sided cineangiography demonstrated a separate, markedly tortuous paraspinal and intercostal collateral network with multilevel segmental filling (Video S2). Together, these findings demonstrated bilateral collateral recruitment involving both intraspinal and extraspinal pathways.

CTA demonstrated severe focal narrowing of the proximal descending thoracic aorta, with a minimum luminal diameter of approximately 9.5 mm, as well as extensive cervicothoracic and intercostal collateralization and diffuse hypoplasia of the left subclavian artery. CoA was diagnosed on the basis of the CTA morphology and extensive collateralization. Upper-lower extremity blood pressure gradient assessment and echocardiography were not performed during the acute phase. Taken together, the CTA and DSA findings supported the interpretation of the ASA aneurysm as a likely flow-related lesion arising in the setting of chronic collateral recruitment. Representative aortic CTA findings are shown in Figure 2.

### 3.4. Treatment

The immediate treatment priority was preventing rebleeding. On the day of presentation, endovascular treatment was performed under general anesthesia through a left common femoral arterial approach using a 6-Fr Envoy DA guiding catheter (Cerenovus, Irvine, CA, USA). Despite the severe stenosis at the coarctation, the guiding catheter was advanced across the stenotic segment and into the right subclavian artery without difficulty. A 45° pre-shaped Excelsior SL-10 microcatheter was further steam-shaped by adding a proximal curve to create a lazy-S configuration. The microcatheter was navigated over a Synchro-2 0.014-inch microwire (both Stryker Neurovascular, Fremont, CA, USA) through the artery of the cervical enlargement and into the aneurysm. The aneurysm was selectively packed with nine detachable coils. Final angiography demonstrated complete aneurysm occlusion with preserved flow through the parent ASA. No intraprocedural or postprocedural complications occurred. The angiographic stages of the endovascular treatment are shown in Figure 3.

Subsequently, C5-6 total laminectomy was performed for cervical spinal cord decompression, together with posterior arthrodesis. This surgery was adjunctive to the preceding coil embolization and was not intended as additional aneurysm-directed treatment. Intraoperatively, the spinal cord appeared swollen, and no definite dural pulsation was observed. Postoperative imaging confirmed satisfactory decompression and instrumentation (Figure 4).

### 3.5. Postoperative Course and Clinical Outcome

By day 20 of her hospitalization, the patient was alert. Her motor strength was 0/5 in the right upper and lower extremities, 3/5 in the left upper extremity (1/5 distal to the wrist), and 3/5 in the left lower extremity. She was discharged on day 23.

At approximately 1-year follow-up, the patient’s left-sided motor function had returned to normal, whereas severe right-sided weakness persisted. She could only bear weight on the right lower extremity with assistance and remained nonambulatory and wheelchair-dependent. At the final follow-up, aortic repair had not yet been performed, as the patient continued to undergo rehabilitation for persistent neurological deficits. Follow-up vascular imaging was not performed; therefore, the long-term durability of aneurysm occlusion could not be assessed.

## 4. Focused Review Results

### 4.1. Scope and Characteristics of the Available Evidence

The general and CoA-focused PubMed searches identified 142 and 12 records, respectively. Eight well-characterized previously published reports contained sufficient patient data for detailed CoA-associated synthesis. Including the present case, nine patients are summarized in Table 3. The available evidence consisted mainly of case reports and small series, and no prospective comparative study of spinal artery aneurysm management was identified.

### 4.2. Morphological and Angiographic Phenotypes

Parent-vessel designations and angiographic appearances varied across the reviewed reports. The ASA was commonly identified as the involved longitudinal arterial axis, particularly at the cervical and thoracic levels; however, some lesions designated as ASA aneurysms arose at radiculomedullary–ASA junctions or were described as dissecting or pseudoaneurysmal [1,2,3,4,5,18,31,36,37]. This variability limited direct comparison across reports [2].

Fusiform morphology was commonly reported among isolated spinal artery aneurysms and may reflect a dissecting spectrum, whereas discrete saccular or multilobulated lesions were also described [2,5]. For descriptive synthesis, angiographic and clinical phenotypes were grouped as saccular or multilobulated; fusiform, dissecting, or pseudoaneurysmal; and giant and/or partially thrombosed (Table 1a). The last category reflects lesion size and thrombus burden rather than a pure morphological class, and overlap among categories was permitted.

### 4.3. Mechanistic Categories and Phenotype–Mechanism Cross-Classification

For descriptive purposes, we developed an author-defined mechanistic framework based on recurring associations reported in the literature. The framework comprised shunt-related lesions; flow-related lesions within recruited collateral pathways, including those associated with CoA or vertebral artery occlusion; and isolated or presumed dissecting lesions [4,7,8,15,18,21,32,38,39]. This framework was used only to organize the literature and has not been validated as a diagnostic or therapeutic classification system. The categories highlight accompanying features that may warrant assessment, including an associated shunt, an obstructive lesion and the full collateral circuit, or a possible dissecting substrate. Table 1a defines the descriptive phenotype and mechanism axes, and Table 1b cross-classifies them using representative reports.

### 4.4. Clinical Presentation and Diagnostic Pathway

Most patients present with SAH, acute neck or back pain, or focal neurological deficits. Cervical lesions are more often associated with infratentorial subarachnoid blood, headache, nausea, and vomiting, whereas thoracic lesions more commonly present concurrently with abrupt back pain [1,2,6,18]. Because the intracranial component of the hemorrhage may be limited or atypically distributed, the initial investigation may focus on a presumed intracranial aneurysm; in some reports, the spinal source was identified only after negative cranial angiography or repeat imaging prompted by neurological deterioration [21,25].

CTA and MRA can detect larger lesions, but catheter spinal angiography is often needed to characterize small aneurysms, dissecting morphology, and complex collateral anatomy [1,2,6,15,18,21,38]. An initially negative angiogram does not exclude a spinal aneurysm, as vasospasm, intraluminal thrombus, or adjacent hematoma may temporarily obscure the lesion. In selected cases, repeat angiography or flat-panel detector CT angiography may assist in identifying an occult lesion or delineating small radicular and radiculomedullary branches [2,21].

### 4.5. Management Strategies and Reported Outcomes

Treatment approaches varied across reports. In the 164-patient synthesis, 47% underwent surgery, 37% were managed conservatively, and 16% underwent endovascular treatment [2]. A separate systematic review of 138 patients reported surgery in 52.1%, observation in 31.1%, and endovascular treatment in 11.5% of cases; in 5.3%, the treatment was not reported [4]. Although microsurgery accounted for approximately half of the published cases in both reviews, these descriptive data do not permit comparison of efficacy among treatment modalities.

Rebleeding was documented in approximately 8% of reported patients overall and at a similar frequency among those managed conservatively; no consistent predictor of rebleeding was identified [2]. Reported mortality was approximately 12%, and clinical deterioration was most often attributed to respiratory complications, spinal cord infarction, and vasospasm [2]. Ultra-early rebleeding before definitive treatment has also been described [26]. Conversely, spontaneous angiographic regression occurred in approximately half of conservatively managed lesions and was significantly associated with a good clinical outcome [2]. Regression has been reported, particularly among small fusiform cervical and thoracic aneurysms [27,28]. These findings support observation with early repeat imaging in selected small fusiform lesions with minimal or improving symptoms, provided that reliable clinical and imaging follow-up is available.

Reported microsurgical techniques included direct clipping or clip reconstruction, trapping or resection, and reinforcement or wrapping [3,4,11,17,19,24,31,32,33,34,38]. The operative corridor varied according to spinal level and the ventral or lateral position of the lesion, ranging from posterior or posterolateral exposure to anterior cervical corpectomy [31]. Intraoperative assessment of parent-vessel tolerance was described using temporary occlusion, Doppler ultrasonography, indocyanine green angiography, and neurophysiological monitoring. In one T4 radiculomedullary artery aneurysm, rapid deterioration of motor evoked potentials during temporary occlusion prompted clip removal and wrapping rather than parent-vessel sacrifice [4,35].

Reported endovascular techniques included selective coil embolization and, in selected fusiform or dissecting lesions, parent-artery occlusion or flow diversion [13,16,20,22,23,29,30]. Across both microsurgical and endovascular approaches, a central consideration was whether the aneurysm-bearing ASA or radiculomedullary artery contributed to spinal cord perfusion or an important collateral pathway and whether parent-artery occlusion could therefore be tolerated. The principal selection considerations are summarized in Table 2.

### 4.6. CoA-Associated Spinal Artery Aneurysms

Recruitment of the spinal arterial axis as a collateral pathway in CoA has been documented angiographically, including flow from cervical radiculomedullary feeders through the ASA to distal segmental arteries and the post-coarctation aorta [11,12,24]. Related non-aneurysmal presentations, including aortic steal-associated neurogenic claudication and compressive myelopathy caused by enlarged tortuous spinal arteries, emphasize the functional importance of this pathway [40,41].

Nine well-characterized patients with sufficient data for detailed tabulation, including the present case, are summarized in Table 3. Most aneurysms involved the cervical or upper thoracic spinal arterial circulation, although one patient had three tandem lesions at T2, T5, and T7 [13]. Seven of the nine patients presented with rupture, most commonly with spinal- or foramen magnum-predominant SAH; other hemorrhagic presentations included hematomyelia and intraventricular hemorrhage with hydrocephalus [13,23,24]. The two unruptured lesions were giant T1 ASA aneurysms presenting with compressive myelopathy [15,17]. CoA was previously unrecognized in two reported patients [22,23] and in the present patient.

Definitive aneurysm management consisted of microsurgery in four patients, endovascular coiling in four, and observation in one. One microsurgically treated patient underwent an unsuccessful endovascular attempt before clipping [24]. Earlier reports predominantly described microsurgical treatment [11,15,17,24], whereas more recent reports included successful coil embolization of both cervical and thoracic lesions [13,22,23]. Outcomes and follow-up were incompletely reported, limiting opportunities for comparison of treatment approaches. Definitive repair of the aortic obstruction was documented in only one patient and was performed after neurological rehabilitation [22].

Upper-limb arterial access was used in two reported cases: an axillary approach for diagnostic angiography [11] and a transbrachial approach for coil embolization [22]. These cases illustrate the potential value of alternative access when the coarctation limits stable catheterization of proximal vessels from a femoral approach.

Additional historical reports and a recent operative video described CoA-associated spinal hemorrhage or spinal/radicular artery aneurysms; however, they lacked sufficient accessible patient-level detail across the prespecified core variables for reliable inclusion in Table 3 [34,42,43,44,45].

## 5. Discussion

This case illustrates a plausible hemodynamic relationship between severe aortic obstruction and aneurysm formation within the spinal arterial circulation. The clinically important finding was not simply the coexistence of CoA and an ASA aneurysm, but the apparent recruitment of the spinal arterial axis as part of an extensive collateral network. Three observations are relevant: the predominance of hemorrhage within the cervical spinal canal provided an early clue to a spinal vascular source; angiography demonstrated a cervical ASA aneurysm in the setting of marked systemic collateralization; and treatment required sequential control of the ruptured aneurysm followed by surgical management of cervical canal compromise. Together, these findings support a flow-related mechanism and emphasize the need to evaluate the aorta and the wider spinal collateral circuit when a spinal artery aneurysm is identified.

### 5.1. The ASA as a Collateral Conduit in CoA

Under normal conditions, the ASA functions as a relatively low-flow longitudinal arterial axis reinforced at variable levels by anterior radiculomedullary arteries. CoA can alter this arrangement by creating a persistent pressure difference between arterial territories proximal and distal to the obstruction, thereby promoting collateral pathways involving the subclavian, internal thoracic, intercostal, vertebral, paraspinal, and intraspinal arterial networks [10,12,46]. In this setting, the ASA may become incorporated into a longitudinal collateral pathway bridging the obstructed aortic segment. In the thoracolumbar region, the ASA receives segmental reinforcement from the great anterior radiculomedullary artery (artery of Adamkiewicz) [47].

Proximal inflow into this spinal collateral circuit may arise from the vertebral, deep cervical, or ascending cervical arteries through a cervical radiculomedullary branch. Blood may flow caudally through an enlarged ASA and continue through lower thoracic radiculomedullary branches into paraspinal or intercostal arteries distal to the coarctation. Flow augmentation or reversal within the spinal arterial system has been demonstrated in patients with CoA even without an aneurysm [11,12,40,41]. The direction and relative contribution of individual vessels are patient-specific and should be determined from selective dynamic angiography rather than inferred solely from vessel caliber.

In this patient, bilateral cineangiography demonstrated longitudinal spinal arterial transit on the left and a complementary tortuous paraspinal and segmental network on the right (Videos S1 and S2). Although these sequences do not quantify flow velocity or pressure, they provide qualitative evidence that collateral recruitment extended beyond the aneurysm-bearing vessel.

Recruitment of the ASA as a major collateral pathway exposes the vessel to a hemodynamic environment that is different from its usual low-flow state. Sustained flow augmentation may cause arterial enlargement and tortuosity, whereas focal acceleration, directional change, or an abrupt transition in vessel caliber may lead to a greater concentration of wall stress. Over time, this may promote focal saccular remodeling, dissection, or pseudoaneurysmal change and predispose the vessel to rupture. ASA aneurysms associated with bilateral vertebral artery occlusion provide an analogous model in which the ASA serves as a collateral route exposed to abnormal flow-related stress [19,20,32].

The multilobulated sidewall morphology of the present aneurysm corresponds to the saccular/multilobulated phenotype in Table 1a, while its occurrence within an apparently recruited ASA collateral pathway supports assignment to the flow-related mechanistic category. Table 1b displays this phenotype–mechanism combination using the present case and representative reports. This categorization is descriptive rather than diagnostic. Nevertheless, the proposed mechanism remains an inference based on vascular architecture rather than direct quantitative measurements of flow or pressure.

The extensive internal thoracic, lateral thoracic, and intercostal collaterals, together with diffuse hypoplasia of the left subclavian artery, support a long-standing aortic obstructive process. CTA demonstrated severe focal narrowing of the proximal descending thoracic aorta consistent with CoA. The proposed hemodynamic mechanism is summarized in Figure 5.

### 5.2. Diagnostic Implications of a Spinal-Predominant Hemorrhage Pattern

The distribution of SAH may provide an early clue to the location of the ruptured lesion. A disproportionate volume of blood within the cervical spinal canal, particularly when intracranial SAH is limited, should raise suspicion of a spinal vascular source. When no intracranial lesion adequately explains the hemorrhage pattern, extending CTA coverage through the cervical spine offers a rapid means of screening for a cervical spinal vascular source.

In the present case, the culprit aneurysm was visible on the initial CTA, which included the cervical spine. Spinal artery aneurysms may otherwise be recognized only after an initially negative intracranial vascular evaluation or after neurological deterioration prompts repeat imaging. Careful review of the foramen magnum and cervical spinal canal on the initial noncontrast CT may therefore shorten the diagnostic pathway when hemorrhage predominantly extends caudally [21].

MRI complements CT and CTA by delineating the craniocaudal extent of SAH, spinal cord edema, hematomyelia, intradural hematoma, and canal compromise. Prominent perimedullary flow voids may also suggest an arteriovenous shunt or an enlarged collateral network.

DSA remains essential for defining aneurysm morphology; the anatomical relationship among the aneurysm, its parent artery, and the ASA; flow patterns; and any associated arteriovenous shunt [1,6,15,18,38]. These findings are particularly important prior to parent-artery occlusion, as an evaluation limited to the aneurysm sac may overlook critical arterial connections and underestimate ischemic risk.

### 5.3. Treatment as Two Sequential Problems

Management addressed two immediate threats: rebleeding from the ruptured aneurysm and neurological injury from cervical spinal cord compression. Because rebleeding could be catastrophic, aneurysm occlusion was prioritized before decompression. No predefined aneurysm diameter threshold was applied; the indication for treatment was the hemorrhagic presentation and the need to prevent rebleeding, rather than aneurysm size alone. The multilobulated sidewall morphology and the feasibility of selective coil embolization with preservation of the parent ASA informed the choice of treatment.

Selective coil embolization preserved the parent ASA and was followed by C5-6 laminectomy and posterior arthrodesis for decompression and stabilization. This sequence reflects the distinction in Table 2 between aneurysm-directed treatment and adjunctive surgery. Given the ventral location of the aneurysm, concurrent microsurgical treatment during posterior decompression would have required additional exposure, with potential manipulation of the spinal cord and parent ASA. Selective coil embolization had already achieved complete aneurysm occlusion with parent-artery preservation, allowing the subsequent surgery to focus on decompression.

Upper-limb access was used in two previously reported CoA-associated cases when vessels proximal to the coarctation were not readily catheterized via a femoral approach [11,22]. In the present patient, CTA showed sufficient luminal caliber at the coarctation for uncomplicated transfemoral catheterization. Access should therefore be individualized according to the stenotic-segment diameter, the aortic arch configuration, and the origin of the aneurysm-feeding artery.

The optimal treatment for an ASA aneurysm depends on phenotype, accessibility, parent-vessel anatomy, and the vessel’s role in spinal or systemic collateral flow. In the present flow-related saccular lesion, selective coiling achieved complete aneurysm occlusion while preserving the parent ASA. Fusiform, dissecting, or pseudoaneurysmal lesions may be managed with reconstruction, parent-vessel occlusion, or observation; when occlusion is considered, angiography should be performed to define the parent vessel’s relationship to the ASA and potential alternative arterial pathways.

Microsurgery offers distinct advantages for surgically accessible lesions, particularly when a large or partially thrombosed aneurysm causes mass effect, hematoma evacuation is required, or endovascular access is not feasible [2,3,4,11,17,19,24,31,32,33,34,38]. When parent-artery occlusion is contemplated, IONM and intraoperative Doppler or ICG angiography can inform decision-making but do not eliminate the technical and ischemic risks associated with the ASA’s small caliber and location on the ventral surface of the spinal cord [4,35].

Despite complete angiographic occlusion and subsequent decompression, the patient had substantial residual neurological disability at approximately 1 year. This outcome illustrates that anatomical treatment success may not translate into functional recovery after neurological injury caused by hemorrhage, ischemia, compression, or the initial cardiopulmonary arrest.

### 5.4. Comparison with Previous CoA-Associated Cases

The rarity of ASA aneurysms and the limited number of CoA-associated cases preclude broad claims of novelty. Table 3 summarizes the heterogeneity of well-characterized reported lesions in spinal level, rupture status, morphology, and management, which has ranged from observation to microsurgical or endovascular treatment [11,13,15,17,22,23,24]. Cervical lesions have previously been treated with both clipping and coiling, and tandem thoracic aneurysms have also been managed endovascularly; this is of particular relevance to the present case [11,13,23,24].

The present case is neither the first reported association between CoA and an ASA aneurysm nor the first CoA-associated ASA aneurysm to be treated endovascularly. Its contribution lies in integrating an unusual presentation with PEA arrest, early recognition of a spinal vascular source, bilateral cineangiographic assessment of the CoA-related collateral network, and staged management of the ruptured aneurysm and cervical cord compression.

## 6. Limitations

In this retrospective single-case report, we were unable to establish a causal relationship between the aortic obstruction, recruitment of the ASA, and aneurysm formation. Although the vascular architecture and bilateral cineangiography support the proposed flow-related mechanism, cineangiography provided only qualitative information on collateral transit. Quantitative flow measurements, trans-stenotic pressure measurements, computational modeling, and histopathological confirmation were unavailable, as was long-term vascular imaging, precluding assessment of aneurysm recurrence or formation of additional lesions.

The literature component was a focused narrative rather than a systematic review and may not have identified every relevant report. Publication bias and incomplete reporting may lead to under-representation of unsuccessful procedures and treatment-related morbidity. Short and inconsistent follow-up, inconsistent separation of angiographic and neurological outcomes, and limited descriptions of aneurysm morphology and collateral anatomy restricted the assessment of durable occlusion, recanalization, the natural history of untreated lesions, and the application of the phenotype–mechanism framework [1,2,4]. These limitations preclude firm conclusions regarding the comparative efficacy of observation, microsurgery, and endovascular treatment.

## 7. Conclusions

A ruptured cervical ASA aneurysm may reveal previously unrecognized CoA and recruitment of the spinal arterial axis as a collateral pathway. Spinal-predominant SAH should raise suspicion of a spinal vascular source and prompt cervical vascular imaging; when prominent spinal or paraspinal collaterals are identified, assessment of the thoracic aorta may also be considered. Treatment selection should be informed by aneurysm phenotype and the vessel’s role in spinal or systemic collateral flow, with the parent artery preserved whenever feasible. When aneurysm treatment and decompression are both required, they may be approached as separate objectives, with sequencing based on rebleeding risk and spinal cord compression.

## Figures and Tables

**Figure 1:**
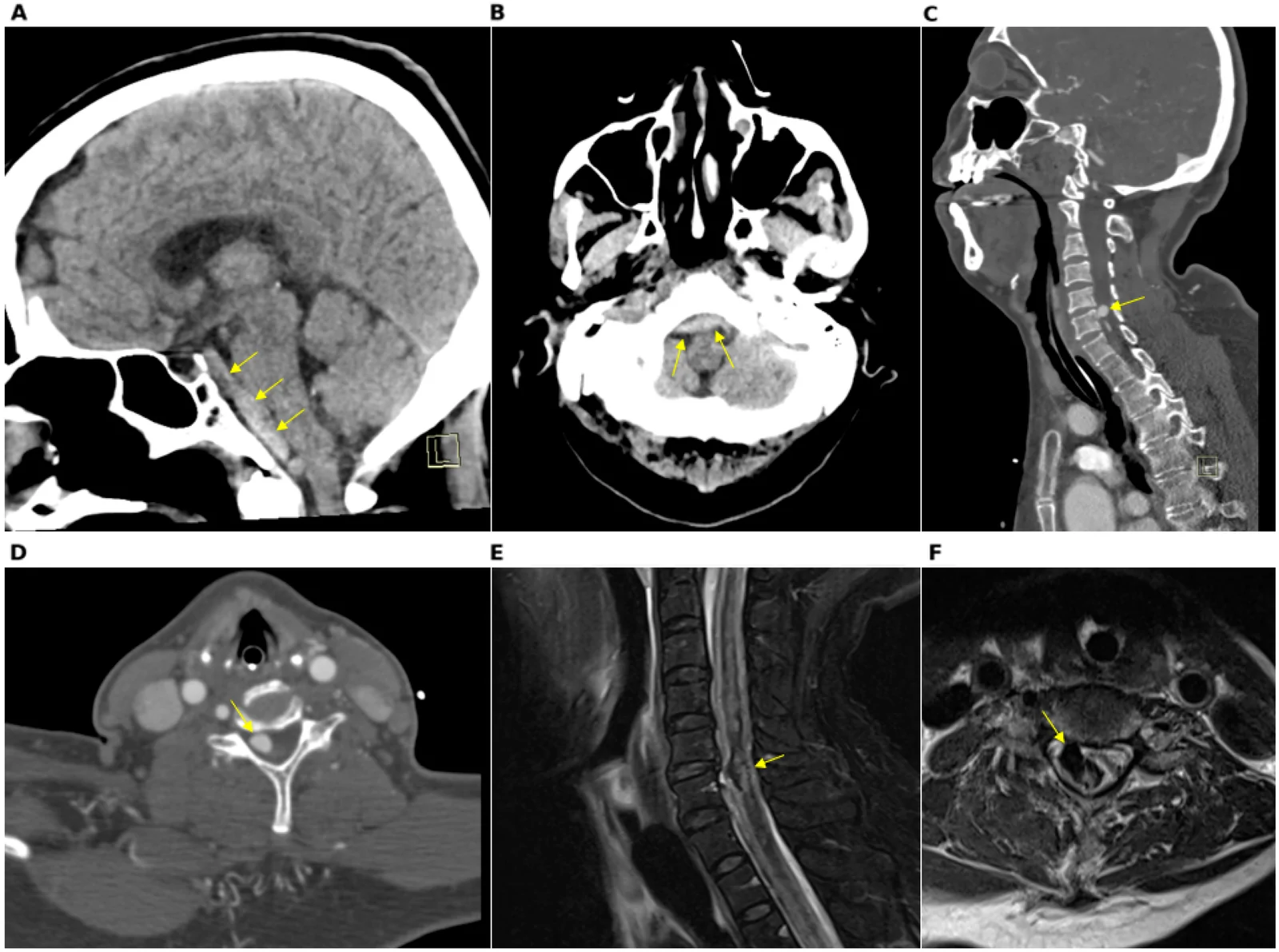
Initial noncontrast CT, cervically extended CT angiography, and cervical MRI. (**A**) Sagittal noncontrast CT demonstrates subarachnoid hyperattenuation extending caudally along the ventral brainstem toward the craniocervical junction (yellow arrows). (**B**) Axial noncontrast CT at the level of the pons shows the relatively limited intracranial component of the subarachnoid hemorrhage within the prepontine cistern (yellow arrows). (**C**) Sagittal source imaging from the extended CTA demonstrates a focal enhancing aneurysmal lesion in the ventral spinal canal at C5-6 (yellow arrow). (**D**) Axial CTA source imaging confirms the enhancing aneurysmal sac in the ventral spinal canal and its close relationship to the spinal cord (yellow arrow). (**E**) Sagittal T2-weighted cervical MRI demonstrates heterogeneous intradural signal extending through the cervical and upper thoracic spinal canal, consistent with extensive spinal subarachnoid hemorrhage, with multilevel effacement of the perimedullary cerebrospinal fluid space; the arrow highlights the focal abnormality at C5-6. (**F**) Axial T2-weighted MRI at C5-6 shows a focal ventral intradural signal void corresponding to the aneurysmal sac (yellow arrow), with heterogenous perimedullary subarachnoid signal and crowding of the spinal cord; no definite intramedullary hemorrhage is identified.

**Figure 2:**
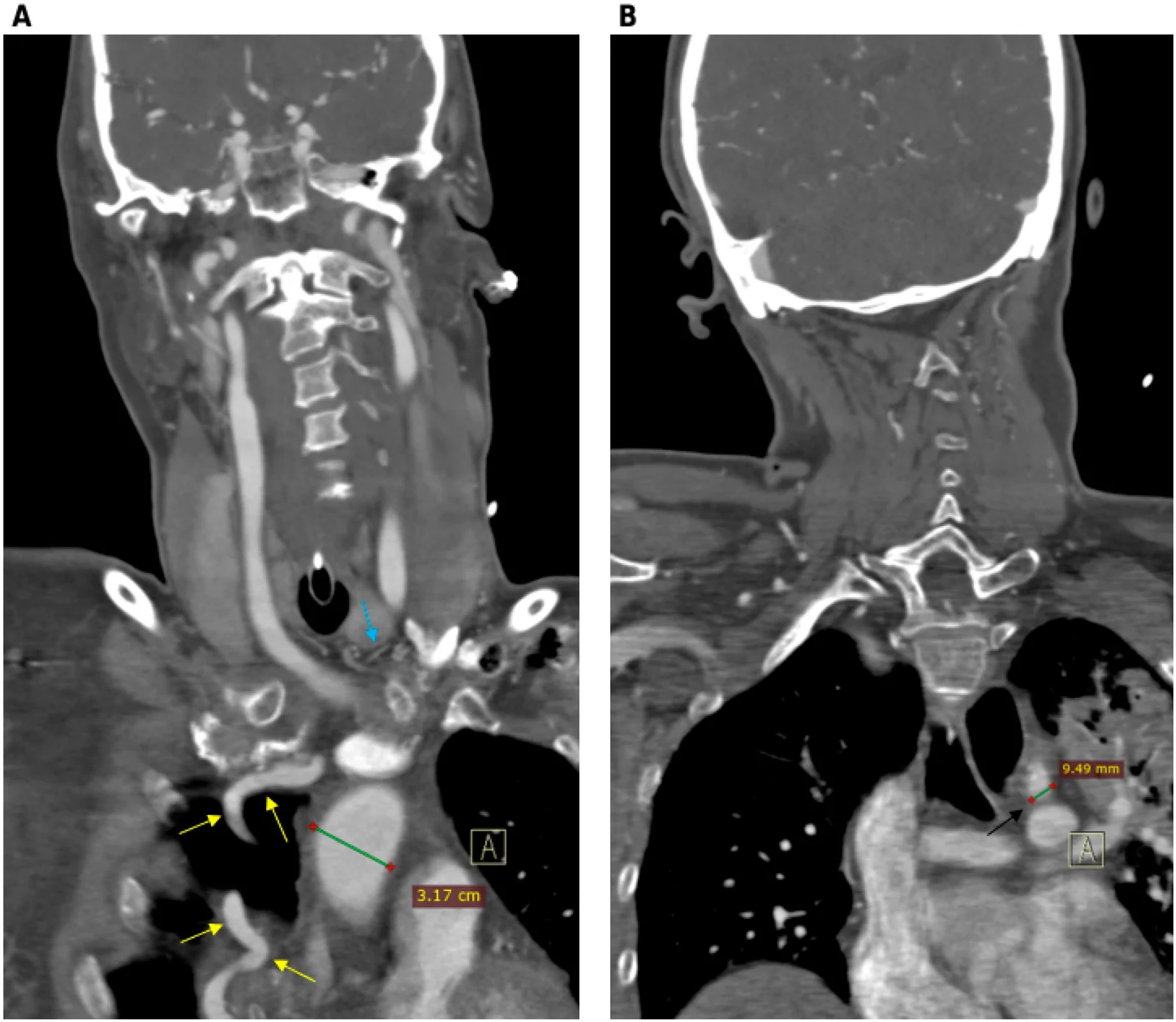
Extended aortic CT angiography demonstrating coarctation of the aorta and systemic collateralization. (**A**) Coronal CT angiographic reconstruction shows prominent right-sided cervicothoracic collateral vessels (yellow arrows) and a portion of the finer left-sided collateral network (blue arrow). The ascending aorta is nondilated, measuring 3.17 cm in diameter, in contrast to the focal narrowing of the proximal descending thoracic aorta shown in (**B**). (**B**) A second coronal reconstruction demonstrates severe focal stenosis of the proximal descending thoracic aorta (black arrow), with a minimum luminal diameter of 9.49 mm. The focal stenotic morphology and extensive collateralization support the diagnosis of CoA.

**Figure 3:**
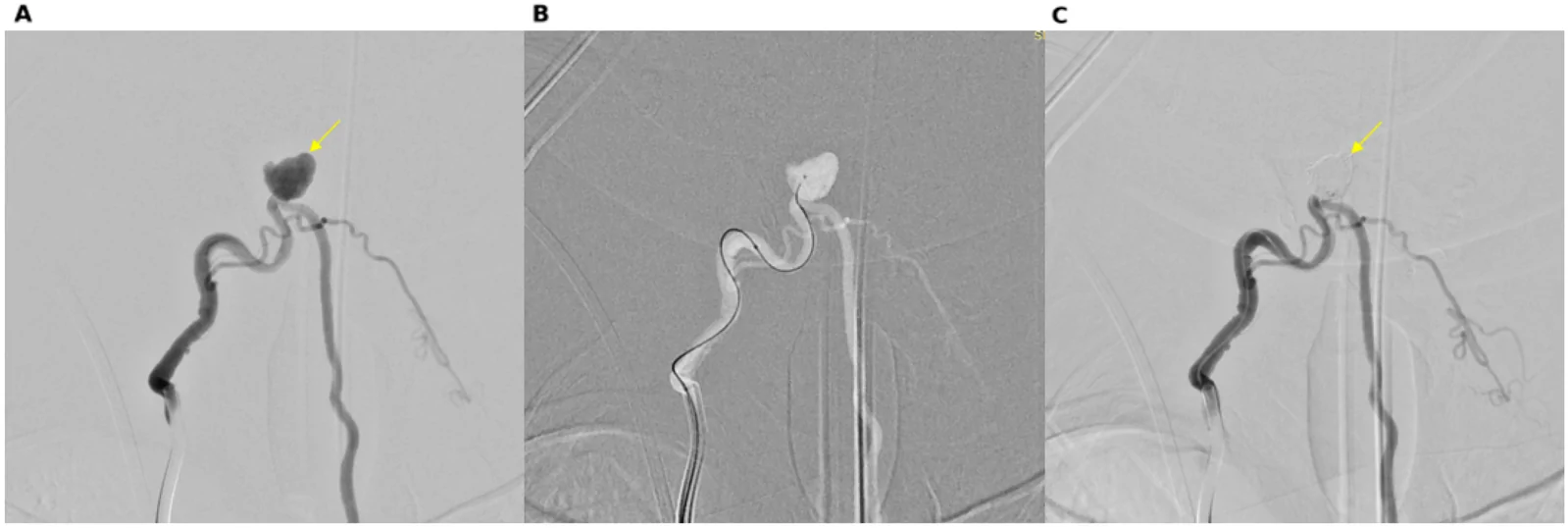
Endovascular coil embolization of the ruptured C5-6 anterior spinal artery aneurysm. (**A**) Selective angiography demonstrates a multilobulated sidewall aneurysm of the cervical anterior spinal artery (yellow arrow). (**B**) Intraprocedural angiography demonstrates selective microcatheterization and progressive coil packing. The nine coils comprised a Target 360 Soft coil (7 mm × 20 cm; framing) and Target 360 Ultra coils (5 mm × 10 cm, 4.5 mm × 10 cm, two 3.5 mm × 8 cm, and 3 mm × 6 cm) (Stryker Neurovascular, Fremont, CA, USA), and Axium Prime 3D coils (6 mm × 20 cm, 4 mm × 10 cm, and 3.5 mm × 6 cm) (Medtronic Neurovascular, Irvine, CA, USA). (**C**) Final angiography demonstrates complete aneurysm occlusion with preserved flow through the parent anterior spinal artery; the yellow arrow indicates the coil mass.

**Figure 4:**
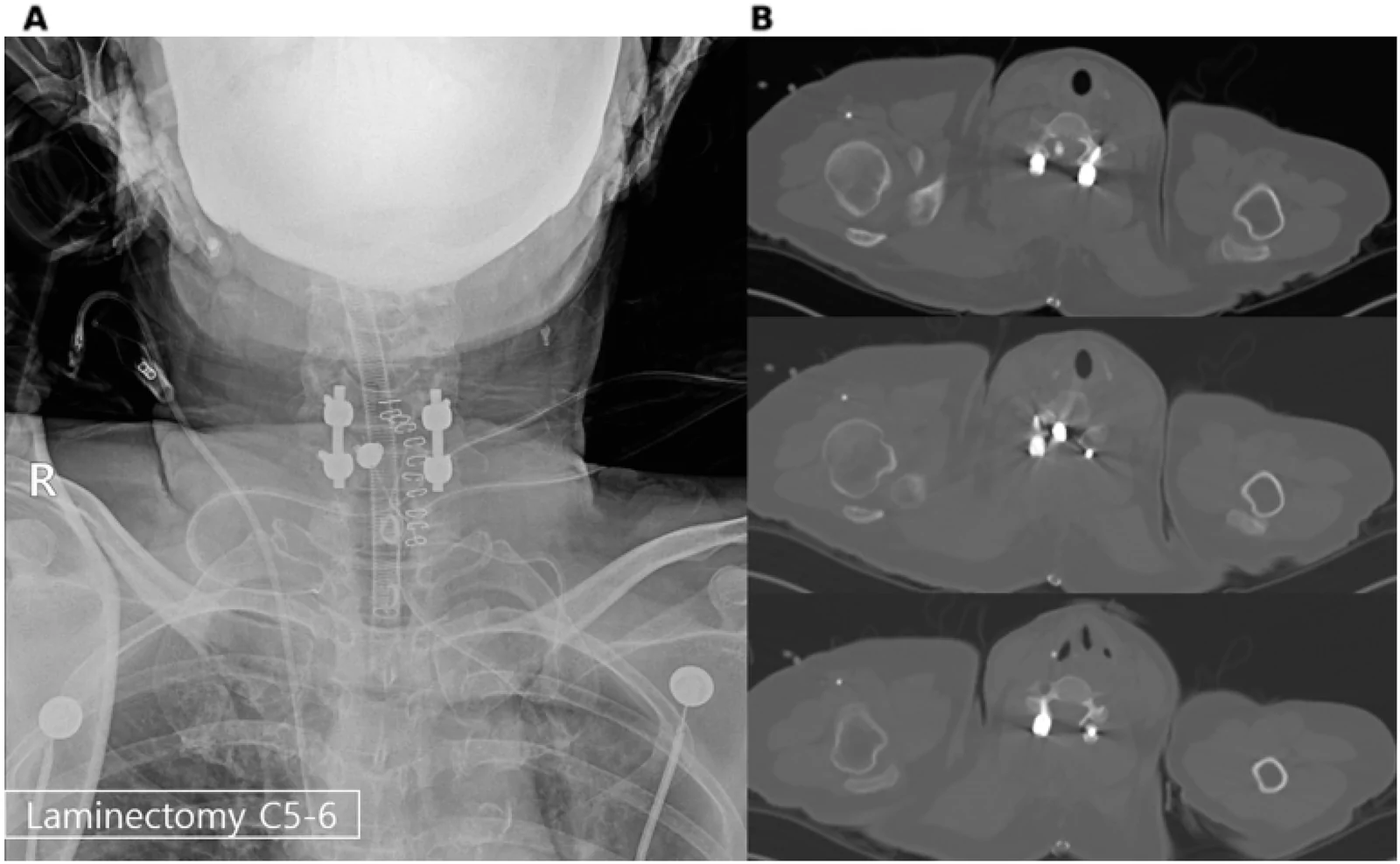
Postoperative imaging after C5-6 decompression and posterior arthrodesis. (**A**) Anteroposterior cervical radiograph shows the bilateral screw–rod construct and coil mass at C5-6. (**B**) Representative axial CT images confirm C5-6 laminectomy with adequate spinal canal decompression and appropriate positioning of the posterior instrumentation.

**Figure 5:**
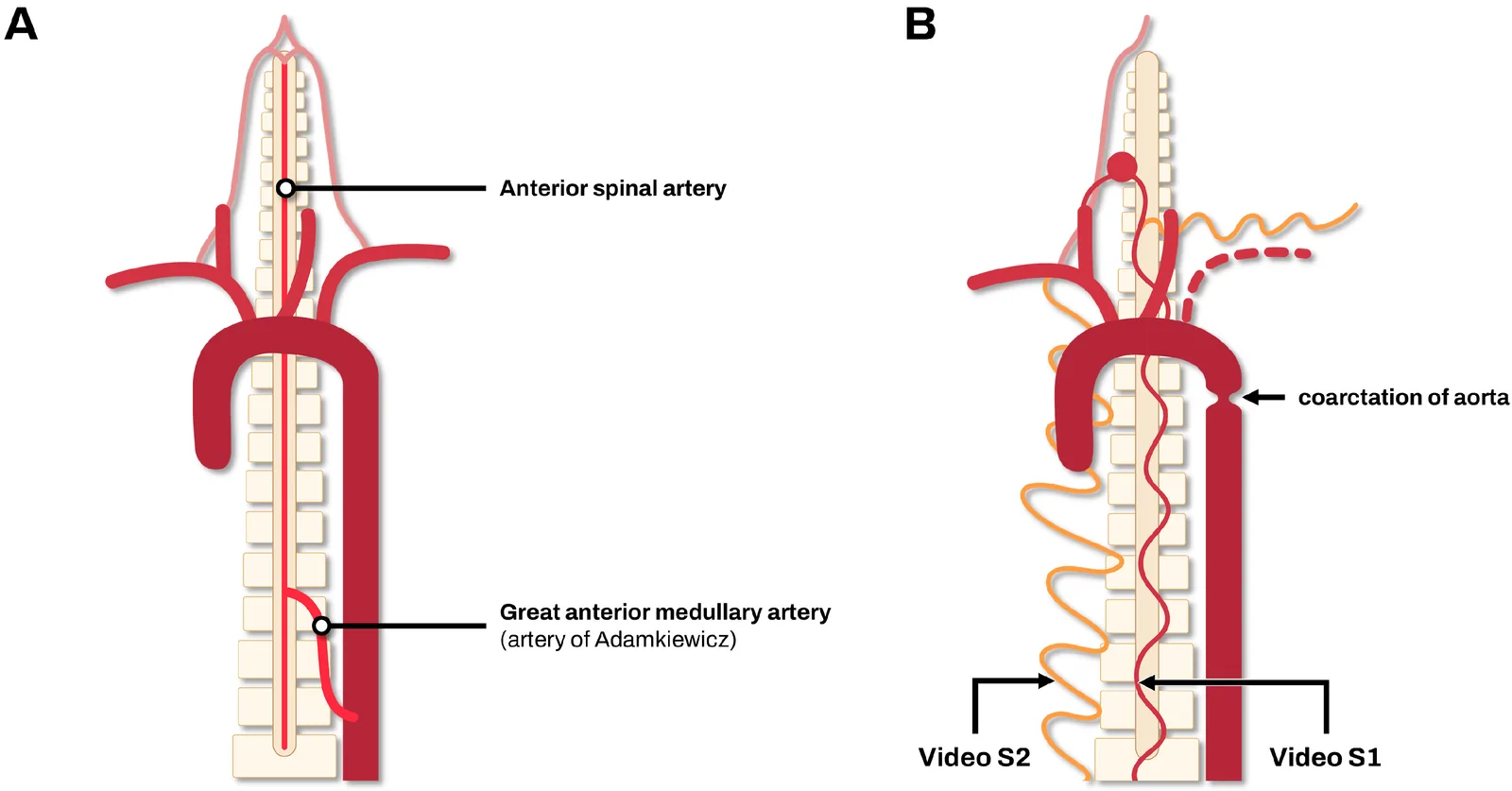
Spinal arterial anatomy and collateral pathways in CoA. (**A**) Normal anatomy. The ASA receives variable segmental reinforcement from anterior radiculomedullary arteries, including the great anterior radiculomedullary artery (artery of Adamkiewicz) in the thoracolumbar region [47]. (**B**) Patient-informed schematic. In this patient, the artery of the cervical enlargement arose directly from the subclavian artery and continued caudally along the anterior spinal cord as the ASA, with the aneurysm located at C5-6. Left-sided cineangiography (Video S1) demonstrated longitudinal transit along the spinal arterial axis, whereas right-sided cineangiography (Video S2) demonstrated paraspinal and intercostal collateralization.

**Table 1:** (**a**) Descriptive classification axes used in the focused narrative synthesis. (**b**) Descriptive cross-classification of angiographic/clinical phenotype and mechanistic category using representative reports.

(**a**)			
**Axis**	**Category**	**Defining Feature**	**Potential Clinical Relevance**
Morphology/Phenotype	Saccular or multilobulated [6,11,15]	Discrete sac or lobules, often with a definable neck arising from a nondilated parent vessel	Selective occlusion with preservation of parent flow may be technically feasible
	Fusiform, dissecting, or pseudoaneurysmal [2,5,16]	Segmental or circumferential dilatation or suspected wall disruption, usually without a discrete neck	Management may range from observation to reconstruction or parent-vessel occlusion depending on lesion stability and perfusion
	Giant and/or partially thrombosed [3,17]	Marked lesion size and/or mural thrombus, with potential mass effect	Mass effect and operative accessibility may become additional considerations
Mechanism	Shunt-related [1,6,18]	An aneurysm arising on a vessel associated with an AVM or AVF, often along a feeding pedicle	Characterization of the shunt and its relationship to the aneurysm may influence treatment selection and sequencing
	Flow-related within a recruited collateral [11,12,15,17,19,20]	Aneurysm arising on an arterial conduit presumed to carry augmented collateral flow across an obstruction, such as CoA or vertebral artery occlusion	Assessment of the full collateral circuit and parent-flow dependence may be relevant before vessel occlusion
	Isolated or presumed dissecting [1,2,5,6]	No associated shunt or major obstructive collateral pathway identified; often fusiform	Lesion evolution, symptoms, and vascular anatomy may inform observation or intervention
(**b**)			
**Mechanistic Category**	**Saccular or Multilobulated**	**Fusiform, Dissecting, or Pseudoaneurysmal**	**Giant and/or Partially Thrombosed Phenotype**
Shunt-related	Chang et al. [21] reported an approximately 3 mm ruptured prenidal ASA aneurysm associated with a cervicomedullary AVM. The lesion was assigned to the saccular category in the present synthesis based on its focal angiographic appearance. *	No representative report was identified in this focused review.	No representative report was identified in this focused review.
Flow-related within a recruited collateral	Jiarakongmun [11] (small lobulated junctional lesion), Aoun [22] (wide-necked saccular lesion), Dang [13] (T2 and T7 saccular lesions), and the present case (C5-6 multilobulated sidewall lesion).	Erdi [23] (C6 dissecting lesion) and Dang [13] (T5 pseudoaneurysm).	Singh case 3 [15] (T1, 18 × 15 mm) and Joseph [17] (T1 giant, partially thrombosed lesion). Both lesions were unruptured, and both patients presented with compressive myelopathy.
Isolated/presumed dissecting	Saccular or discrete-necked isolated lesions have been reported [1,2].	A pooled analysis reported fusiform morphology in 82% of 208 isolated spinal artery aneurysms [2].	No representative report of an isolated giant and/or partially thrombosed lesion was identified in this focused review.

Note: (**a**) Categories may overlap, and “giant and/or partially thrombosed” denotes a clinical size-and-thrombus phenotype rather than a pure morphological category. References are representative rather than exhaustive. This author-defined framework was developed for descriptive synthesis and has not been validated as a diagnostic or therapeutic classification system. Abbreviations: AVF—arteriovenous fistula; AVM—arteriovenous malformation; CoA—coarctation of the aorta. (**b**) Cells contain representative reports rather than an exhaustive enumeration. Phenotype assignments were based on the angiographic and clinical descriptions and, where available, published images in the original reports; they reflect the authors’ interpretation for the purposes of this focused synthesis. Categories may overlap, and “giant and/or partially thrombosed” represents a size-and-thrombus phenotype rather than a mutually exclusive morphological category. * Chang et al. [21] described the lesion as a ruptured prenidal aneurysm without assigning a specific morphological category; its classification as saccular in this table reflects the authors’ interpretation of its focal angiographic appearance. Sharma [24] and Singh case 1 [15] are included in Table 3 but have not been assigned to a phenotype column because their morphology was not reported in sufficient detail. “No representative report identified” indicates that no qualifying example was found in this focused review and does not establish the absence of that phenotype–mechanism combination. This author-defined framework has not been validated as a diagnostic or therapeutic classification system. Abbreviations: ASA—anterior spinal artery; AVM—arteriovenous malformation.

**Table 2:** Management strategies and principal selection considerations for spinal artery aneurysms.

Strategy	Potentially Suitable Setting	Reported Techniques	Main Advantage	Principal Limitation
Observation	Selected small, stable, often fusiform/dissecting lesions with minimal or improving symptoms and reliable follow-up	Close neurological observation with short-interval CTA, MRA, or DSA	Avoids procedural manipulation and treatment-related ischemia; spontaneous regression may occur	Rebleeding or enlargement is difficult to predict, making reliable follow-up essential [2,25,26,27,28]
Endovascular treatment	Discrete saccular lesions with accessible necks; selected fusiform or dissecting lesions when parent-artery occlusion is considered tolerable	Selective coiling; parent-artery occlusion; selected flow diversion or liquid embolization	Provides access to ventral or deep lesions without direct spinal cord manipulation	Small vessel caliber, tortuosity, access difficulty, inadvertent branch occlusion, and uncertain durability [13,16,20,22,23,29,30]
Microsurgical aneurysm treatment	Surgically accessible lesions; mass effect or associated hematoma; failed or infeasible endovascular access; need for direct reconstruction	Clipping or clip reconstruction; trapping or resection; wrapping, with Doppler ultrasonography, ICG angiography, temporary occlusion, or IONM as indicated	Allows direct visualization and reconstruction and can be combined with hematoma evacuation or decompression	Ventral location, spinal cord manipulation, and potential interruption of critical ASA or radiculomedullary flow [3,4,11,17,19,24,31,32,33,34,35]
Decompression or hematoma evacuation (adjunct)	Mechanical spinal cord compression, intradural or intramedullary hematoma, or instability requiring fixation	Laminectomy or corpectomy; hematoma evacuation; stabilization when required	Addresses mechanical neural injury and mass effect	Does not secure the aneurysm unless combined with aneurysm-directed surgery; timing must be individualized

ASA—anterior spinal artery; CTA—computed tomography angiography; DSA—digital subtraction angiography; ICG—indocyanine green; IONM—intraoperative neurophysiological monitoring; MRA—magnetic resonance angiography. This author-defined framework summarizes reported management practices and practical selection considerations; it is not a validated or comparative treatment algorithm. Decompression or hematoma evacuation is listed separately because it addresses neural compression rather than the aneurysm itself unless combined with aneurysm-directed surgery.

**Table 3:** Patient-level characteristics of well-characterized published spinal arterial aneurysms associated with CoA and the present case.

Study	Age/Sex	CoA Characteristics	Level and Lesion Phenotype	Presentation/Rupture	Aneurysm Treatment	Additional Spinal/Aortic Treatment	Outcome
Jiarakongmun [11]	39/M	CoA	Cervical RM–ASA junction; small lobulated aneurysm	Neck pain, SAH, quadriparesis	Microsurgical clipping	No CoA repair described	Not stated
Sharma [24]	36/M	CoA	C6 ASA aneurysm supplied by enlarged RM artery	Hematomyelia, quadriparesis, sphincter dysfunction; ruptured	Endovascular coil embolization unsuccessful microsurgical clipping	No CoA repair described	Lower-limb power 4/5; near-normal upper-limb and sphincter function at 4 months
Aoun [22]	59/F	Previously undiagnosed isthmic CoA	7 mm wide-necked saccular ASA aneurysm	SAH	Endovascular coil embolization	Interposition-graft CoA repair after rehabilitation	Returned to asymptomatic neurological baseline
Erdi [23]	55/F	Previously unrecognized CoA	C6 dissecting spinal artery aneurysm, 10 mm	Tetraparesis; intraventricular hemorrhage and hydrocephalus	Endovascular coil embolization	External ventricular drainage	Not stated
Singh, case 1 [15]	18/M	Proximal descending CoA	C7 ASA aneurysm, 5.6 × 3.3 mm	Spinal SAH	Observation	None described	Not stated
Singh, case 3 [15]	46/M	CoA	T1 giant ASA aneurysm, 18 × 15 mm	Compressive myelopathy; unruptured	Microsurgical clipping	None described	Not stated
Joseph [17]	50/M	CoA	T1 giant, partially thrombosed ASA aneurysm	Chronic compressive myelopathy; unruptured	Microsurgical clipping	No CoA repair described	Early renal failure and pulmonary edema resolved by discharge
Dang [13]	54/M	CoA	T2 and T7 saccular lesions; T5 pseudoaneurysm	Foramen magnum SAH; T5 considered the culprit	Endovascular coil embolization at T5 and T2; untreated at T7	No CoA repair reported	Lower-limb strength improved from grade I to II at 15 days
Present case	58/F	Thoracic CoA with left subclavian artery hypoplasia	C5-6 multilobulated sidewall ASA aneurysm, 9.8 × 6.9 mm	PEA arrest; spinal-predominant SAH	Endovascular coil embolization	Decompressive C5-6 laminectomy	Left-sided recovery; severe right-sided weakness at 1 year; wheelchair dependence

ASA—anterior spinal artery; CoA—coarctation of the aorta; F—female; M—male; PEA—pulseless electrical activity; RM—radiculomedullary; SAH—subarachnoid hemorrhage. Only cases with sufficient data for detailed tabulation are included. All cases were considered flow-related within a recruited collateral. Jiarakongmun [11] was classified as lobulated on the basis of the original report. Sharma [24] and Singh case 1 [15] were not assigned to a Table 1b phenotype cell because the morphological descriptions were insufficient. Additional treatment is separated from aneurysm-directed therapy.

## Data Availability

The data supporting the findings of this case report are available from the corresponding author on reasonable request, subject to patient privacy and institutional restrictions.

## Supplementary Materials

The following supporting information can be downloaded at https://www.mdpi.com/article/10.3390/jcm15197364/s1, Video S1. Left-sided collateral spinal angiography. Cineangiography demonstrates sequential opacification of an enlarged cervical radiculomedullary vessel and longitudinal spinal arterial channel, with caudal progression toward lower thoracic segmental outflow. File: Lt_collateral_spinal_angiography.mp4; duration: 9.4 s; playback rate: 15 frames/s. Video S2. Right-sided collateral spinal angiography. Cineangiography demonstrates a markedly enlarged, tortuous paraspinal and intercostal collateral network with multilevel segmental filling. File: Rt_collateral_spinal_angiography.mp4; duration: 14.2 s; playback rate: 15 frames/s.
